# Supplementary material for: Reconstruction of the unbinding pathways of noncovalent SARS-CoV and SARS-CoV-2 3CLpro inhibitors using unbiased molecular dynamics simulations
Source: PLoS One. 2022 Feb 9;17(2):e0263251. doi: 10.1371/journal.pone.0263251 (PMC8827461; doi:10.1371/journal.pone.0263251)
Supplement: Attachment — Submitted filename: Response to Reviewers.docx [file pone.0263251.s008.docx]

**Dear Prof. Chandrabose Selvaraj;**

I am pleased to mail you the revised article titled **"Reconstruction of the unbinding pathways of noncovalent SARS-CoV and SARS-CoV-2 3CLpro inhibitors using Unbiased Molecular Dynamics"**. All comments have been answered upon reviewers' recommendations, and the corrections were made in manuscript #PONE-D-21-32120. It is our pleasure to hear your feedback and any other suggestions relevant to the paper.

**Sincerely,**

**Hassan Aryapour**

**Reviewer #1:**

**Comment #1:**

Capitalization of abbreviations "SARS-CoV" and "SARS-CoV-2" needs to be corrected in the title, as well as in the text. Additionally, capitalization of other words, such as "viruses" in the abstract needs to be corrected.

**Answer #1:**

The requested items were corrected.

**Comment #2:**

Also, there seems to be an issue with font type and size which is inconsistent.

**Answer #2:**

The entire text was reviewed and corrected.

**Comment #3:**

Throughout the manuscript, English should be corrected e.g. the sentence (lines 35-36): "Coronaviruses (CoVs), which have the largest genome among RNA viruses, are enveloped by single-stranded RNA viruses." does not make any sense.

**Answer #3:**

A new version of this sentence is highlighted in (lines 40-42).

**Comment #4:**

The sentence (lines 71-72): "On the other hand, investigation of the unbinding pathways of particular inhibitors, protein-ligand key interactions, and ligand's effectiveness or weakness can be obtained." needs to be rephrased, it does not make a lot of sense.

**Answer #4:**

A new version of this sentence is highlighted in (lines 85-87).

**Comment #5:**

The sentence (line 73): "Ultimately, the obtained results will be presented as a fully atomistic scenario [17]." needs to be rephrased (and font changed).

**Answer #5:**

A new version of this sentence is highlighted in (lines 88-89).

**Comment #6:**

The sentence (lines 86-90): "Since, half-maximal inhibitory concentration (IC50) of SARS-COV 3CLpro inhibitors are measured experimentally before, and the 3CLpro of SARS-COV and SARS-CoV-2 are approximately the same, our research team for participating in the global solidarity trial decided to examine some noncovalent SARS-CoV and SARS-CoV-2 protease inhibitors' unbinding pathways by the SuMD, Comparatively." is very confusing and needs to be rephrased. Also, what does "approximately the same" even mean? Additional clarification is needed and does this influence the binding site?

**Answer #6:**

This paragraph was rewritten for better understanding. We used "approximately the same", based on multiple sequence alignment, which reported 96.08% identity and 98.7% similarity between 3CLpro of SARS-CoV and SARS-CoV-2. There is no difference for the 3CLpro binding sites of these two viruses, so we added this information to the new version of this paragraph.

**Comment #7:**

There is no discussion and comparison of the obtained results with results of other papers, have other researchers performed similar studies, are the conclusions the same, is there any difference in results or a new insight?

**Answer #7:**

PDB IDs 3V3M and 4MDS have other research, and we added a comparison of their results with ours in the result and conclusion sections in the (lines 187-190 & 240-242 & 371-375) but for the two other PDB IDs (6W63 and 5RF7) there are no articles.

**Comment #8:**

What are the differences between the SARS-CoV and SARS-CoV-2 3CLpro binding sites? How does this reflect on binding of the studied inhibitors and their selectivity? If there are not any differences, it should also be said.

**Answer #8:**

The comment was answered alongside Comment #6.

**Comment #9:**

In figures, such as Figure 3 l, m and n, should the word "state (S)" be "bound state"?

**Answer #9:**

In all figures, state words were replaced with bound states (B).

**Comment #10:**

Figures in general are of low quality, I don't know if this is a consequence of converting to the PDF format or not.

**Answer #10:**

We fixed the problem of low-quality figures caused by PDF conversion.

**Comment #11:**

Can the authors explain the "free energy" term in e.g. Figure 2 m, n, o, is it the ∆GBIND or something else?

**Answer #11:**

The free energy landscape plots were obtained using "gmx sham" as we mentioned in the figures. Actually, "gmx sham" leads to plot Gibbs's free energy landscapes.

**Comment #12:**

Can the authors also explain how come in some cases (e.g. Figure 3.c) the total interaction energy goes as low as -350 kJ/mol, what does this interaction energy represent?

**Answer #12:**

Total interaction energies plots are obtained from the sum of the coulomb and Lennard-jones contribution values. We have maximum interaction energies in the bound state of the protein-ligand complex. As time passes and protein-ligand interactions weaken, the values of the plot increase and eventually reach zero until full unbinding. So all the interactions energies plots' trend is correlated with the unbinding process from the bound state to the unbound state. Also, -350 kJ / mol values in some plots and -300 kJ / mol values in others indicate the value of bound state interaction energies. Since these interactions measure in vacuum conditions, these values cannot prove that the inhibitor with a lower interaction energy value in a bound state is more potent. These plots should be examined along with the average of the most important interaction energies for better understanding.

**Comment #13:**

In line 255, what does the expression "the almost deep part" mean?

**Answer #13:**

The word "almost" is deleted.

**Comment #14:**

In the Conclusion, English is especially poor: the sentence (lines 336-338): "All fragments of inhibitors did not interact seriously at specific residues in the binding pocket, so this factor was sufficient to weaken critical interactions, resulting in rapid unbinding." is very confusing and has no meaning.

**Answer #14:**

A new version of this sentence is highlighted in (lines 358-361).

**Comment #15:**

The same goes for the sentence after that (lines 338-341): "These almost free fragments could compete with other fragments for interactions with essential residues. Even if they did not enter into competitive interactions, they could change inhibitors' position and brought the inhibitor closer to the exit path by their rotations." which needs to be rephrased.

**Answer #15:**

A new version of this sentence is highlighted in (lines 361-365).

**Comment #16:**

The sentence (lines 353-355): "In conclusion, the next series of noncovalent inhibitors should be designed so that they can occupy all S regions of the binding pocket to make maximum noncovalent interactions." Does not say anything new, this is common knowledge – the inhibitors should fill out the binding site as much as possible. Are there any concrete suggestions the authors have for development of future 3CLpro inhibitors, which interactions are good and should stay, which interactions are bad and should be corrected etc.?

**Answer #16:**

We explained everything, even common knowledge, because we wanted to report all atomistic details. Moreover, we explained all favorable and unfavorable interactions in the results and discussion sections for all inhibitors' unbinding processes. The most potent interactions are also listed in the conclusion section (lines 367-370).

**Reviewer #2:**

**Comment #1:**

The methodology of this work is correct, the analysis is well supported, and the results are partially interesting.

**Comment #2:**

The SuMD MD simulation is interesting to unbind the inhibitor from the active site because the center of masses distance is increased in each window. Perhaps, for pure classical Molecular Dynamic Simulations, just 500 ps could be not enough to equilibrate the system. Have the authors tried to increase the simulation time by window to avoid possible equilibration problems?

**Answer #2:**

Equilibration was applied by performing 100 ps of NVT and 300 of NPT. The 500 ps simulation is not for equilibration. The method section has been improved for better understanding.

**Comment #3:**

The structure of the paper and the results are very clear. There are four systems: two inhibitors and two proteins (3CL of SAR-CoV and SARS-CoV-2). For each system, three replicas unbinding the inhibitor (from 25 to 65 ns). For the results, same figures and same analysis to compare the results properly. My major criticism about this paper is that the main goals from the results: on one hand, when the inhibitor is unbinding, water molecules are coming in the active site, so, when the inhibitor occupy the S region, the binding should be better because less water molecules are affecting the inhibitor-enzyme interaction, so the main idea is that "water molecules played a functional role in all unbinding mechanisms by interfering and breaking important protein-inhibitor interactions", which is something very well known in from many other works On the other hand, the inhibitor interacts such as independent fragments with different parts of the proteins. I think these findings are not very novel, however, some results are very interesting such as Figures 2, 4 and 6, parts m, n and o. These maps should be explained by the authors more carefully.

**Answer #3:**

For the "On the other hand, the inhibitor interacts such as independent fragments with different parts of the proteins. I think these findings are not very novel" we explained in the answer of **Comment #16** of reviewe1. Also, we added a more detailed explanation of the Free energy landscape plots in the method section.

**Comment #4:**

The bibliography is insufficient. There are many QM/MM Molecular Dynamic simulations and classical simulations of many inhibitors of the 3CL enzyme of SAR-CoV and SARS-CoV-2 which must be included by the authors.

**Answer #4:**

The QM/MM Molecular Dynamic simulations are a hybrid method that is entirely unrelated to our work. Our study is MM Molecular Dynamic simulations, which there is no related project to mention.

**Comment #5-1:**

The structure of the inhibitors should be provided in the introduction section. In addition, the structure of the active site of the complex protein-inhibitor highlighting the critical distances should be showed as well.

**Answer #5-1:**

All fragments of each inhibitor are existence in the figures of the result section. If we change the location of these pictures, the readership of this article may get board to switch to the introduction section and again back to the result section. The active site domain of the protein is explained in Figure1, and a binding pocket of the complex protein-inhibitor is picturizing separately in Figures 2, 4, 6, and 8.

**Comment #5-2:**

Page 9, line 178 "j and k" looks like a typo mistake

**Answer #5-2:**

This line was rechecked.

**Comment #5-3:**

Footnote of Figure 3. Cut-offs are 0.35 and 0.5 angstroms? I think there is a mistake is those data.

**Answer #5-3:**

True values of Cut-offs were replaced in figures' captions.

**Reviewer #3:**

**Comment #1:**

In the abstract, the author provided more general info instead of the results of the study. In addition, the conclusion and methods are not appropriate---make it clear.

**Answer #1:**

More information about the conclusion is added in the abstract section. Also, the Conclusion and method sections were improved.

**Comment #2:**

In the introduction section, the author might start with SARS first, then SARS-2 to follow the uniformity of the title of the study.

**Answer #2:**

The requested information was added in the introduction section (lines 31-32)

**Comment #3:**

Page 8 – lines 46-49: "The domains I and II have 46 antiparallel β barrel structures responsible for the catalytic reaction, while domain III has α-helices (Fig 1a, b). Since the dimeric form of Mpro is catalytically active, intermolecular interactions between the helical domains play an essential role in activating the enzyme [8, 9]. ------ It is not clear. What is the role of domain III?

**Answer #3:**

The requested information was added in the introduction section (line 53)

**Comment #4:**

What is unbiased molecular dynamics (UMD) and its advantages? Brief up 1 or 2 lines.

**Answer #4:**

The requested information was added in the introduction section (lines 75-77)

**Comment #5:**

This line seems to be meaningless – "MD simulation has been a standard method of predicting hidden information in many science fields since the 1950s"----what is meant by hidden info by MD simulations?

**Answer #5:**

Since the experimental research gives information based on x-ray crystallographic binding pose, results are statics and limited, but MD simulations are dynamic and give information about what is happening during unbinding pathways. Therefore, this information cannot be obtained through experimental research and is regarded as hidden.

**Comment #6:**

What is the correlation of the above lines and "in judging candidate drugs, the bioavailability, selectivity, metabolic properties, and binding affinity of the designed inhibitor to its target protein are all important? [16].”

**Answer #6:**

For better understanding, we have rewritten this sentence (lines 81-84).

**Comment #7:**

It is not an appropriate word "ligand's effectiveness or weakness can be obtained". It may be ligands interacting efficiently with the target

**Answer #7:**

The "ligands interacting efficiently with the target" was replaced with "ligand's effectiveness or weakness can be obtained" in (lines 86-87).

**Comment #8:**

These lines are a bit confusing or meaningless "Since, half-maximal inhibitory concentration (IC50) of SARS-COV 3CLpro inhibitors are measured experimentally before, and the 3CLpro of SARS-COV and SARS CoV-2 are approximately the same, our research team for participating in the global solidarity trial decided to examine some noncovalent SARS-CoV and SARS-CoV-2 protease inhibitors' unbinding pathways by the SuMD, Comparatively." From MD authors defining IC50 value?

**Answer #8:**

Experimental research provided the IC50 value. The reference is added in (line 104).

**Comment #9:**

This is unclear: we performed 12 simulations, three replicas for each complex, by the SuMD method with some modifications---what are 12 simulations? Whether author's starts from 1ps to 500ps or 1ps to 1500 ps defined as replicas? Because the following line authors mentioned, "------to extend the next 500 ps simulation".

**Answer #9:**

We improved the method section. Actually, we ran all simulations with a time window of 500 ps. We mentioned in the method section, "After finishing each run, the frame with the longest distance between selected spots was selected automatically to extend the next 500 ps simulation". These processes were continued until complete unbind was obtained, which is equal to a distance of 50 Å between the mentioned spots", So each replica is a separate simulation from binding pose to complete unbinding.

**Comment #10:**

Reference missing for UCSF Chimera

**Answer #10:**

The reference is available in (line 113).

**Comment #11:**

Author may improve the discussion because in the results and discussion section authors described only results.

**Answer #11:**

The conclusion section changed to improve.

**Comment #12:**

How do the authors create a free energy plot? Which package?

**Answer #12:**

The requested information was added in the method section (lines 144-150).

**Comment #13:**

In all the figures, authors may reduce the thickness of the lines to get better visualization---figures are not in good resolution.

**Answer #13:**

All figures were rebulided again, but for more precise images, we reduced the thickness of the lines, resulting in plots that faded

**Comment #14:**

Conclusion should be improved.

**Answer #14:**

The conclusion section changed to improve.

**Comment #15:**

The language of the manuscript should meet the journal's adequate standard.

**Answer #15:**

The requested work is done.
