# Supplementary material for: Reconstruction of the unbinding pathways of noncovalent SARS-CoV and SARS-CoV-2 3CLpro inhibitors using unbiased molecular dynamics simulations
Source: PLoS One. 2022 Feb 9;17(2):e0263251. doi: 10.1371/journal.pone.0263251 (PMC8827461; doi:10.1371/journal.pone.0263251)
Supplement: Attachment — Submitted filename: Response to Reviewers.docx [file pone.0263251.s009.docx]

**Dear Prof. Chandrabose Selvaraj;**

I am pleased to mail you the revised article titled **"Reconstruction of the unbinding pathways of noncovalent SARS-CoV and SARS-CoV-2 3CLpro inhibitors using Unbiased Molecular Dynamics"**. All comments have been answered upon reviewers' recommendations, and the corrections were made in manuscript #PONE-D-21-32120R1. It is our pleasure to hear your feedback and any other suggestions relevant to the paper.

**Sincerely,**

**Hassan Aryapour**

**ACADEMIC EDITOR:**

You may want to upload a letter of editing from a professional agency or native-English speaker in addition to the reviewer's comments because reviewers have expressed a concern about the language part.

**Answer:**

The entire document has been reviewed by someone who has a better understanding of English and checked for existing problems in the text

**Journal Requirements:**

Please review your reference list to ensure that it is complete and correct. If you have cited papers that have been retracted, please include the rationale for doing so in the manuscript text, or remove these references and replace them with relevant current references. Any changes to the reference list should be mentioned in the rebuttal letter that accompanies your revised manuscript. If you need to cite a retracted article, indicate the article's retracted status in the References list and also include a citation and full reference for the retraction notice.

**Answer:**

The references list was reviewed once more carefully, and no reference was changed.

**Reviewer #1:**

The authors have significantly improved the manuscript and added further discussion of their results. There are still significant language issues that need to be corrected throughout the manuscript (with e.g. lines 39-40 "Coronaviruses (CoVs), an RNA virus with the largest genome, are enveloped by a single-stranded RNA virus. "– coronaviruses are enveloped RNA viruses, they are not enveloped BY a single stranded RNA virus; and lines 99-101: "So our research team, for participating in the solidarity clinical trial for COVID-19 treatments, decided to examine some noncovalent SARS-CoV and SARS-CoV-2 protease inhibitors' unbinding pathways by the SuMD, Comparatively. "which is still grammatically very incorrect). Additionally, my comment about the lack of discussion and comparison with results from other researchers is still standing. The authors have improved their discussion, but they have obtained a lot of new information which could have been more thoroughly discussed and compared, it would further increase the significance and value of their work. Nonetheless, even in the present state, I believe that this research should be accepted for publishing.

**Answer #2:**

The lines 39 and 40 and also lines 99-101 were replaced with the corrected version in (lines 39-40 and 111-114).

For the comparison, our research method is different from the mentioned experimental research. They examined important protein-inhibitor interactions only in a binding pose, so we could compare only their ultimate result with a little part of our work.

**Reviewer #2: 1) Answer #5-1:**

Reviewer #2: 1) Answer #5-1:

All fragments of each inhibitor are existence in the figures of the result section. If we change the location of these pictures, the readership of this article may get board to switch to the introduction section and again back to the result section. The active site domain of the protein is explained in Figure1, and a binding pocket of the complex protein-inhibitor is picturizing separately in Figures 2, 4, 6, and 8.

Comment#5-1:

I still think that a scheme of the inhibitors in the introduction section would increase the clarity of the study.

**Answer:**

Inhibitors' structure image was replaced in the introduction section (Fig2 and lines 62-69).

**2) Answer #3:**

For the "On the other hand, the inhibitor interacts such as independent fragments with different parts of the proteins. I think these findings are not very novel" we explained in the answer of Comment #16 of reviewe1. Also, we added a more detailed explanation of the Free energy landscape plots in the method section.

From the free energy landscapes, would be possible to predict a free energy of binding value of each system?

**Answer:**

For calculating binding free energy, there are some ways involving: alchemical transfer method (ATM), linear interaction energy (LIE), PDLD/S-LRA method, alchemical free energy perturbation (FEP), BAR method, and so on. But, We computed the free energy landscape (FEL) to capture inhibitors' lowest energy stable states during unbinding by gmx sham module (https://manual.gromacs.org/documentation/2018/onlinehelp/gmx-sham.html).

This module calculates free energy landscapes by computing the joint probability distribution from the two-dimensional plane constructed using two quantities (in our case, they were ligand RMSD (as the x-axis) and protein RMSD (as the y-axis)). Conformations sampled during the simulation were projected on this two-dimensional plane, and the number of points occupied by each cell was counted. The grid cell containing the maximum number of points is then assigned as the reference cell, with a free energy value of zero. Free energies for all the other cells were assigned with respect to this reference cell using the following equation:

ΔG(x,y) = -KbT ln P(x,y)/Pmin

P(x,y) estimates the probability density function obtained from a histogram of MD data, and Pmin is the maximum of the probability density function. Kb is the Boltzmann constant, and T is the temperature corresponding to each simulation.

**3):** The initial Molecular Dynamic Simulations should be longer to obtain an equilibrated configuration of the proposed models. Before SuMDs, the system should be very well equilibrated. You can see many studies about 3CL enzyme in which classical MD of microsecond timescales is performed before to study binding or reaction processes. The initial protocol of this work does not allow, perhaps, to explore important configurations of the enzyme that should be considered in the study. Authors should explore this way carefully for future studies.

**Answer:**

Thanks for your valuable advice, and we will consider them for our future research.
